# Unraveling the Molecular Mechanisms of Glioma Recurrence: A Study Integrating Single‐Cell and Spatial Transcriptomics

**DOI:** 10.1002/acn3.70306

**Published:** 2026-01-06

**Authors:** Lei Qiu, Yinjiao Fei, Jiaxuan Ding, Kexin Shi, Jinyan Luo, Yuchen Zhu, Xingjian Sun, Gefei Jiang, Yuandong Cao, Weilin Xu, Shu Zhou

**Affiliations:** ^1^ Department of Oncology, Beijing Tiantan Hospital Capital Medical University Beijing China; ^2^ Department of Oncology, Sir Run Run Hospital Nanjing Medical University Nanjing Jiangsu China; ^3^ Department of Radiation Oncology, Sun Yat‐Sen Memorial Hospital Sun Yat‐sen University Guangzhou Guangdong China; ^4^ Department of Radiation Oncology The First Affiliated Hospital with Nanjing Medical University Nanjing Jiangsu China; ^5^ The First Clinical Medical College of Nanjing Medical University Nanjing Jiangsu China

**Keywords:** glioma, recurrence, single‐cell RNA sequencing, spatial transcriptomics

## Abstract

**Objective:**

Glioma recurrence severely impacts patient prognosis, with current treatments showing limited efficacy. Traditional methods struggle to analyze recurrence mechanisms due to challenges in assessing tumor heterogeneity, spatial dynamics, and gene networks. Single‐cell combined spatial transcriptomics (ST) offers innovative solutions.

**Methods:**

We analyzed glioma mRNA data from TCGA and single‐cell and ST data from GEO. Following quality control, dimensionality reduction, clustering, and cell annotation of single‐cell sequencing data, we identified cell types exhibiting significantly aberrant distributions between primary and recurrent samples by analyzing the deviation degree of Ro/e values. Fibroblasts demonstrating the greatest intergroup differences were subsequently selected as the key cellular population for further investigation. Key differentially expressed genes (DEGs) were identified via random survival forest analysis. Drug sensitivity was assessed using GDSC. Deconvolution algorithms mapped cellular spatial distribution, while PROGENy quantified pathway activity. MISTy modeling revealed cell–cell interactions.

**Results:**

Fibroblasts were the primary recurrence‐associated subpopulation, with marker genes enriched in extracellular matrix and adhesion pathways. AEBP1, ZNF708, and TSHZ2 were identified as key genes: AEBP1/TSHZ2 correlated with poor prognosis, while ZNF708 showed an inverse trend. These genes were linked to chemosensitivity (Irinotecan, Carmustine, Vincristine, and Cisplatin). Recurrent tumors exhibited increased plasma cell infiltration, with key genes regulating IL‐17, Notch, and Toll‐like receptor pathways. Spatial analysis highlighted oligodendrocyte‐astrocyte interactions in the tumor microenvironment.

**Interpretation:**

Fibroblasts drive glioma recurrence, with AEBP1, ZNF708, and TSHZ2 predicting recurrence and chemoresistance. These genes promote immune suppression (via plasma cells) and activate recurrence pathways. Oligodendrocyte‐astrocyte interactions shape the recurrent microenvironment, suggesting new therapeutic targets.

## 1 Introduction

Gliomas represent the most prevalent type of primary brain tumors, making up 81% of malignancies in the central nervous system (CNS) [1]. Due to the highly aggressive nature of gliomas, achieving complete surgical removal is often challenging, contributing to subsequent patient relapse [2]. In particular, high‐grade glioma (HGG) patients exhibit recurrence rates as high as 90% [3]. Even in cases of low‐grade gliomas (LGG), approximately 60% will progress to HGG upon recurrence [4]. The elevated recurrence rate of gliomas is a critical factor impacting patient prognosis. Currently, there is no standardized treatment protocol or consensus for recurrent gliomas. A combination of surgery, radiotherapy, chemotherapy, TTFields, and targeted therapy is generally regarded as the most appropriate treatment approach [5]. These therapies are also constrained by factors such as the patient's functional status, age, extent of tumor resection, and drug resistance, resulting in a median overall survival of only 6–15.5 months following recurrence [6, 7, 8, 9]. Therefore, it is imperative to develop new therapeutic strategies to enhance patient outcomes.

The evolution of bioinformatics has been instrumental in advancing cancer research, enabling the systematic discovery of molecular biomarkers and therapeutic targets from high‐throughput genomic data. Numerous studies have successfully leveraged bulk transcriptomic and epigenomic profiles to identify clinically relevant signatures, as exemplified by the identification of ABCG1 hyper‐methylation as a diagnostic biomarker in non‐small cell lung cancer and the comprehensive bioinformatics analysis implicating GPSM family members in breast cancer pathogenesis [10, 11]. The underlying molecular and genetic basis of gliomas has evolved in recent years, opening up possibilities not only for early diagnosis of the disease but also for new strategies for individualized treatment. Key prognostic markers include IDH mutation status, 1p/19q codeletion, MGMT promoter methylation, as well as TP53 and EGFR alterations [12, 13, 14]. At present, the research on gene changes and survival prognosis of glioma is gradually deepening, but the exploration of gene expression of glioma recurrence is still unclear. Establishing a clear correlation between genetic alterations and glioma recurrence requires more research to explore.

The introduction of single‐cell RNA sequencing (scRNA‐seq) has made it possible to examine the heterogeneity of tumor cells and to recognize different cellular subpopulations and their functional conditions at the level of individual cells [15]. However, the execution of single‐cell sequencing necessitates the dissociation of cells from their tissue context, resulting in the loss of spatial information regarding cellular localization. Spatial transcriptomics (ST) represents a technique that integrates imaging, biomarker analysis, sequencing, and bioinformatics, making it an ideal approach for elucidating the heterogeneity and spatial distribution of cancer cells within tissues [16]. The combination of scRNA‐seq and ST allows for the simultaneous acquisition of single‐cell resolution and spatial positional data, thereby providing a more comprehensive perspective for investigating the mechanisms underlying the occurrence or development of a tumor [17]. By systematically integrating scRNA‐seq and ST, Liu et al. uncovered that MDK‐NCL‐mediated immunosuppression drives endometrial carcinoma progression, proposing pathway blockade as a therapeutic strategy [18]. He et al. utilized scRNA‐seq and ST to investigate the role of oxidative stress response genes in glioma oligodendrocyte progenitor cells (OPCs) [19]. Despite significant technological progress in other types of cancer, there is limited understanding of how scRNA‐seq combined with ST can be used to investigate the molecular mechanisms and genetic factors underlying glioma recurrence.

Consequently, the objective of this research was to develop a scRNA‐seq integrated ST in order to investigate the molecular mechanisms underlying glioma recurrence.

## 2 Materials and Methods

### 2.1 Data Download

The Cancer Genome Atlas (TCGA) database (https://portal.gdc.cancer.gov/) represents the most extensive repository of cancer‐related genetic information [20]. For this study, we downloaded the original mRNA expression data for glioma, gathering a total of 170 samples, which include 157 from the primary tumor group and 13 from the recurrent tumor group.

The GEO database (https://www.ncbi.nlm.nih.gov/geo/info/datasets.html) is a comprehensive gene expression omnibus established and overseen by the National Center for Biotechnology Information (NCBI) in the United States [21]. We can access the NCBI GEO public database to download the GSE174554 single‐cell data file, which comprises a total of 73 samples with complete single‐cell expression profiles, including 31 primary tumor samples and 42 recurrent tumor samples. Additionally, the GSE270355 spatial transcriptome data file is available for download, containing complete spatial transcriptome expression profiles from four glioma samples for spatial transcriptome analysis.

### 2.2 Quality Control

The expression profile was initially imported utilizing the Seurat package, which facilitated the filtering of cells based on several criteria, including the total number of unique molecular identifiers (UMIs) per cell, the number of expressed genes, and the percentage of mitochondrial and ribosomal reads per cell [22]. Outliers were identified as three median absolute deviations (MAD) from the median. The filtering formula employed was as follows: (nFeature_RNA > 200 & percent.mt ≤ median + 3MAD & nFeature_RNA ≤ median + 3MAD & nCount_RNA ≤ median + 3MAD & percent.ribo ≤ median + 3MAD). In this context, nFeature_RNA denotes the number of genes, nCount_RNA indicates the total number of UMIs within the cell, percent.mt refers to the proportion of mitochondrial reads, and percent.ribo signifies the proportion of ribosomal reads. It is widely acknowledged that cells exhibiting excessively high percentages of mitochondrial and ribosomal reads are indicative of compromised quality, potentially approaching apoptosis or existing as cellular debris.

### 2.3 Data Normalization

Initially, the dataset underwent normalization through the application of the NormalizeData function. Following this, cell cycle scores were computed utilizing the CellCycleScoring method, and highly variable genes were identified via the FindVariableFeatures function. Subsequently, the data were scaled using the ScaleData function to reduce the impact of mitochondrial genes, ribosomal genes, and cell cycle effects on subsequent analyses. Linear dimensionality reduction was then executed on the expression matrix through the RunPCA function, with principal components selected for further examination. Harmony is an algorithm specifically designed for the integration of single‐cell RNA sequencing data. To address batch effects, the Harmony algorithm was employed, which iteratively clusters similar cells from different batches within PCA space while maintaining the diversity of batches within each cluster [23, 24]. Nonlinear dimensionality reduction was subsequently performed using Uniform Manifold Approximation and Projection (UMAP) through the RunUMAP function. Cell neighborhoods were delineated using the FindNeighbors function, and cells were categorized into distinct clusters via the FindClusters function. Finally, cell types and their corresponding marker genes present in the relevant tissues were identified by consulting the CellMarker database and relevant literature as the primary approach, supplemented by automatic annotation through the SingleR software for cell classification.

### 2.4 Random Survival Forest

The Random Survival Forest is a machine learning algorithm utilized for the analysis of survival data [25]. In this study, the random survival forest algorithm was implemented using the randomForestSRC package to screen the feature genes and rank the importance of prognosis‐related genes. Prior to constructing the random survival forest model, all candidate differentially expressed genes (DEGs) were subjected to univariate Cox proportional hazards regression analysis (with a significance threshold of *p* < 0.05). This step substantially reduced the dimensionality of the input variables, thereby mitigating the risk of overfitting from the outset. A total of 1000 iterations were then performed in the Monte Carlo simulation (nrep = 1000). To identify the most reliable predictive genes, we applied a relatively conservative threshold (relative importance > 0.2) to select the final key genes.

### 2.5 Drug Sensitivity Analysis

Utilizing the most extensive pharmacogenomics database, the GDSC Cancer Drug Sensitivity Genomics Database (accessible at https://www.cancerrxgene.org/), we applied the R package “pRRophetic” to predict the chemotherapy sensitivity of individual tumor samples. The IC50 values for each chemotherapy agent were calculated through regression analysis, with the GDSC training set subjected to 10 cross‐validation iterations to evaluate the accuracy of regression and prediction. All parameters were maintained at their default settings, including the “combat” function to mitigate batch effects and the averaging of repeated gene expression data.

### 2.6 Immune Infiltration

The CIBERSORT methodology represents a significant analytical framework utilized for the evaluation of immune cell types within various microenvironments [26]. This approach is grounded in the principles of support vector regression and conducts deconvolution analysis on the expression matrix of immune cell subtypes. It encompasses 547 biomarkers and differentiates 22 distinct human immune cell phenotypes, which include T cells, B cells, plasma cells, and various myeloid cell subsets. In this study, the CIBERSORT algorithm was employed to analyze the sample data, enabling the inference of the relative proportions of the 22 immune infiltrating cell types and facilitating correlation analyses between gene expression and immune cell composition.

To quantify the infiltration levels of immune and stromal cells in tumor samples, we applied the Estimation of STromal and Immune cells in MAlignant Tumor tissues using Expression data (ESTIMATE) algorithm. Using the filterCommonGenes function from the estimate package, we filtered the gene expression matrix and subsequently applied the estimateScore function to calculate immune scores, stromal scores, and ESTIMATE combined scores for each tumor sample. This algorithm infers the cellular composition of tumor tissues at the overall transcriptome level by analyzing the expression enrichment of predefined immune‐ and stromal‐related gene signatures. The resulting scores were used in subsequent analyses to explore associations between the tumor microenvironment and clinical phenotypes.

### 2.7 GSEA Analysis

Based on the expression levels of key genes within the samples, the samples were classified into high and low expression groups. Following this classification, the differences in signaling pathways between the two groups were examined utilizing Gene Set Enrichment Analysis (GSEA). The background gene set utilized for this analysis was the version 7.0 annotation gene set obtained from the MsigDB database, which served as the annotation gene set for the subtype pathway. A differential expression analysis of pathways between the distinct groups was conducted, and the significantly enriched gene sets (with an adjusted *p* value of less than 0.05) were ranked according to their consistency scores. GSEA is commonly employed to explore the complex relationship between disease classification and biological significance [27].

### 2.8 GSVA Analysis

Gene set variation analysis (GSVA) is a non‐parametric, unsupervised method for evaluating the enrichment of transcriptome gene sets [28]. This approach converts alterations at the gene level into modifications at the pathway level by methodically scoring the relevant gene sets, thereby clarifying the biological functions linked to the samples being analyzed. In this study, gene sets will be downloaded from the Molecular signatures database, and the GSVA algorithm will be employed to systematically score each gene set, facilitating an assessment of the potential changes in biological functions across different samples.

### 2.9 Spatial Domain Identification

The raw UMI count matrix, imaging data, image coordinates, and scaling factors were imported into R utilizing the Seurat package. The raw UMI counts were normalized through regularized negative binomial regression employing the SCTransform method. Linear dimensionality reduction was performed on the top 3000 genes that displayed the most pronounced variations in expression levels, which were classified as highly variable genes, through principal component analysis (PCA). Following this, nonlinear dimensionality reduction was executed using the RunUMAP function. Clustering was ultimately accomplished through the application of the FindClusters function.

### 2.10 RCTD Deconvolution

Robust cell‐type decomposition (RCTD) is a supervised learning method that decomposes RNA sequencing mixtures into individual cell types, enabling the assignment of cell types to spatial transcriptomic pixels [29]. Its maximum likelihood model is particularly robust in handling technical differences between platforms, making it highly suitable for this task [30]. In particular, we use annotated scRNA‐seq data to define cell type‐specific profiles of cell types expected to be present in spatial transcriptomic data. A related challenge of supervised cell type learning is the phenomenon known as the platform effect, which refers to the influence of technology‐dependent library preparation on the capture rate of individual genes between sequencing platforms. The research indicates that neglecting platform effects may hinder the success of supervised methodologies, as systematic technical variability tends to overshadow the pertinent biological signal. However, RCTD effectively mitigates platform‐specific effects and is applicable for deconvolution analysis of spatial transcriptomes across various platforms [31, 32, 33]. RCTD demonstrates a significant level of accuracy in identifying the spatial localization of cell types within both simulated and actual spatial transcriptome datasets.

### 2.11 Spatial Cell Interactions

Multiview Intercellular SpaTial modeling framework (MISTy) facilitates a deeper understanding of marker interactions by analyzing intracellular and intercellular relationships [34]. It facilitates the development of models that characterize various spatial contexts, particularly concerning the relationships identified in marker expressions, including intracellular and exocrine regulation. For each relationship type, MISTy incorporates a model component referred to as a “view.” These views can encapsulate functional relationships, such as pathway activity and crosstalk, relationships specific to particular cell types, or interactions among different anatomical regions. Subsequently, the contributions of each view to the overall expression of each marker are analyzed. The assessed contributions highlight the correlations among potential sources of interactions arising from diverse spatial contexts and are estimated through view‐specific models. This method has been applied in colorectal cancer, where MISTy confirmed a particular reliance of NUhighepi on fibroblasts [35].

### 2.12 Statistical Analysis

All statistical analyses were conducted using R software (version 4.3.0), with a significance threshold set at *p* < 0.05.

## 3 Results

### 3.1 Quality Control

**FIGURE 1:**
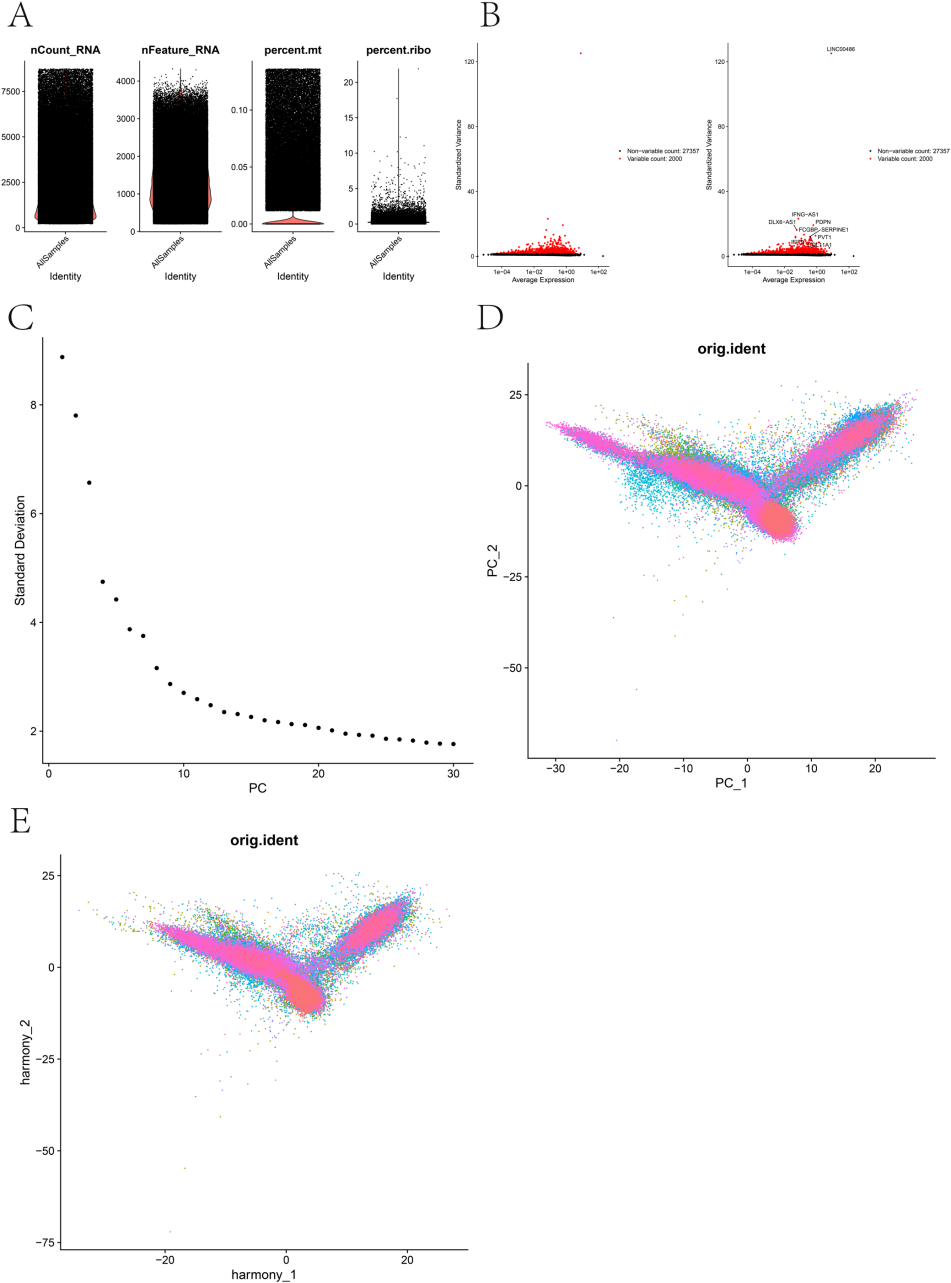
Preprocessing of single‐cell data. (A) Quality control visualization of single‐cell RNA sequencing data. Each subplot displays the number of genes, the number of Unique Molecular Identifier detected per cell (UMI count), the proportion of mitochondrial genes (%MT), and the proportion of ribosomal genes (%Ribo). The *x*‐axis represents the samples, and the *y*‐axis represents the corresponding metric values. (B) Volcano plot of differentially expressed genes. The *x*‐axis represents the log fold change in gene expression between samples, and the *y*‐axis represents the significance of differential expression (−log10(*p*‐value)). Red dots indicate significantly differentially expressed genes (FDR < 0.05), with labeled genes being biologically relevant candidates. (C) ElbowPlot of principal component analysis (PCA) results. The *x*‐axis represents the number of principal components, and the *y*‐axis represents the proportion of variance contribution of each principal component. This plot is used to select the number of significant principal components. (D) Visualization of PCA results. A two‐dimensional scatter plot of the first two principal components (PC1 and PC2). Each point represents a cell, with different colors indicating different cell clusters. (E) Revisualization of double PCA results after adjustment. The colors and point distributions are updated to explore the correction effects of potential batch effects or other technical biases.

Following comprehensive quality assessment across multiple samples, cells with fewer than 200 detected genes were filtered out, retaining 136,677 high‐quality cells for downstream analysis (Figure 1A). We identified 2000 highly variable genes (Figure 1B) and subsequently performed data normalization, scaling, PCA, and batch correction using Harmony (Figure 1C–E). By comparing the clustering patterns of cells based on their sample origins before and after integration, we confirmed that Harmony effectively mitigates technical variations while preserving biological differences.

### 3.2 Data Standardization and Cell Annotation

**FIGURE 2:**
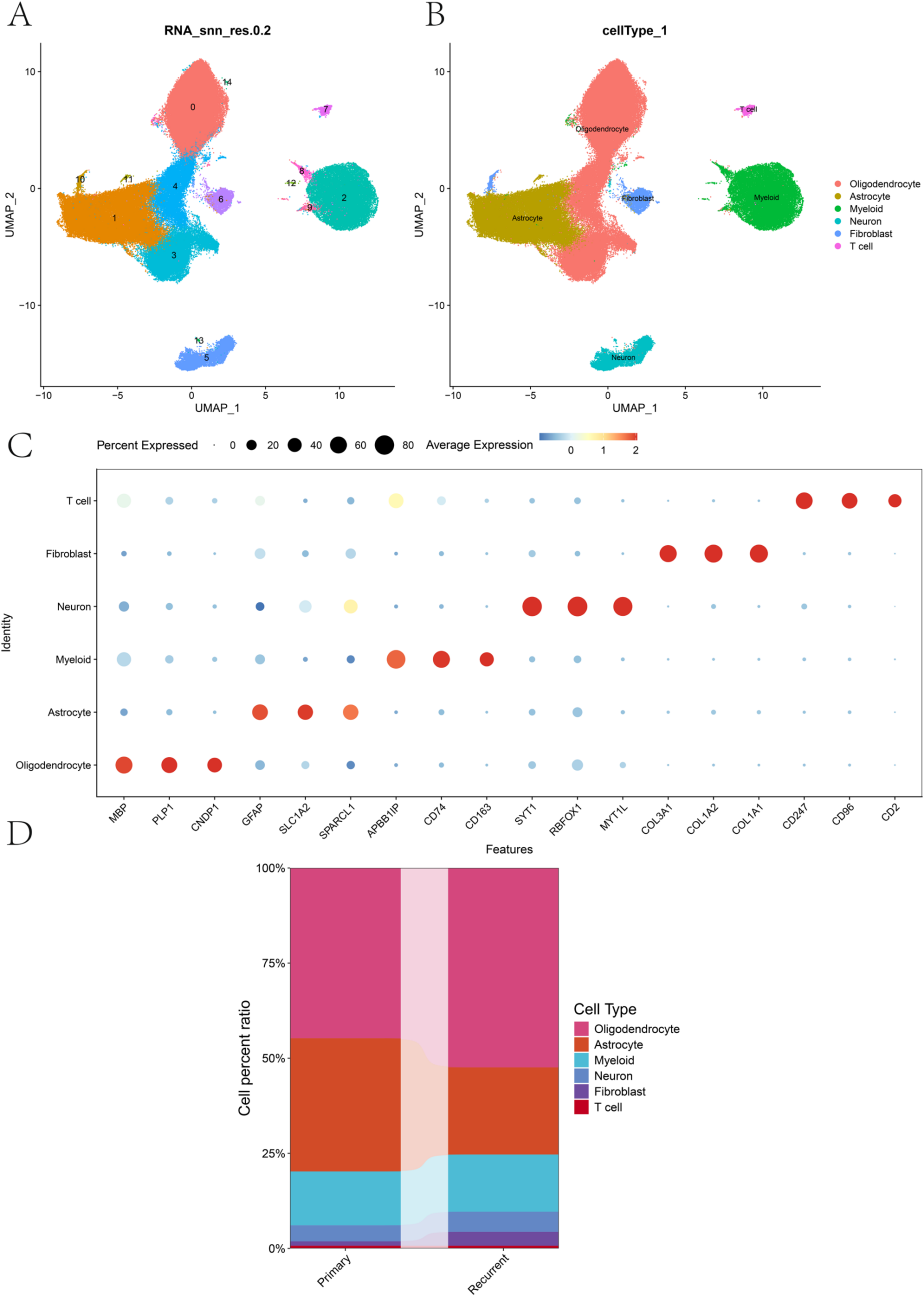
Cell annotation. (A) UMAP plot showing cell clustering results (based on RNA expression, resolution = 0.2). Each point represents a cell, with colors distinguishing different clusters. The *x*‐axis and *y*‐axis represent UMAP coordinates, indicating cellular heterogeneity. (B) UMAP plot showing cell type annotation results. Based on clustering results, cells are further annotated into different cell types, such as Neuron, Fibroblast, and T cell. Different colors and labels indicate the distribution of different cell types. (C) Bubble plot showing the expression patterns of marker genes across different cell types. The *x*‐axis represents genes, and the *y*‐axis represents cell types. The size of the bubbles indicates the proportion of cells expressing the gene in the corresponding cell type (% expressed), and the color represents the average expression level (red for high expression, blue for low expression). (D) Proportional bar charts illustrating the percentage distribution of the six cell types.

**FIGURE 3:**
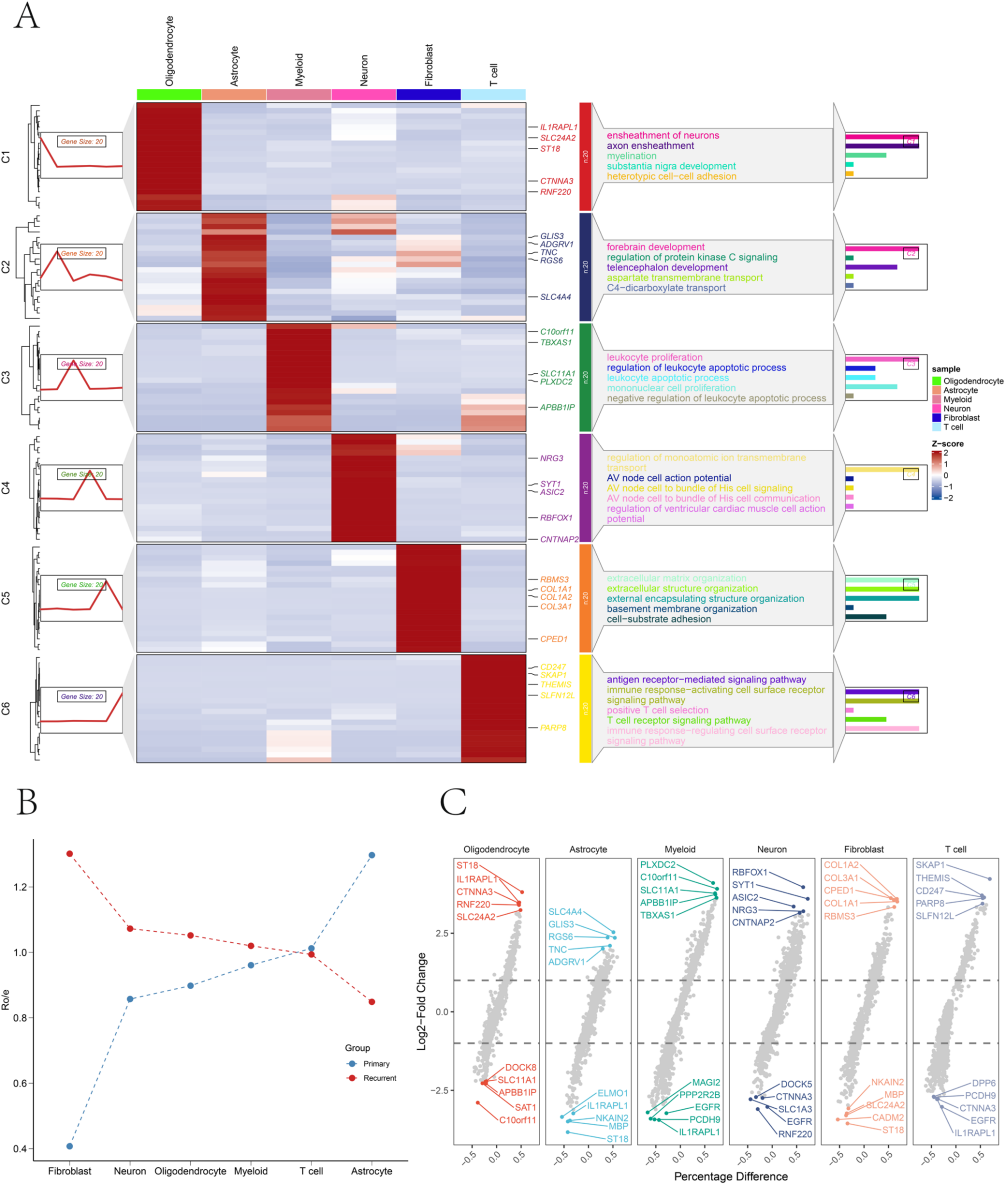
Subpopulation differences and enrichment analysis. (A) Heatmap showing the expression levels of various genes across different cell types. Each row represents a specific gene, and each column represents a cell type. The color scale indicates the *Z*‐score of gene expression, ranging from −2 (blue, low expression) to 2 (red, high expression). The heatmap also includes annotations of biological processes related to the genes. (B) Specificity analysis of cell type distribution in primary and recurrent glioma samples. The ratio of observed to expected values (Ro/e) for each cell type in primary (blue) and recurrent (red) groups is calculated. Cell types are arranged in ascending order by the Ro/e value in the primary group, with dashed lines connecting data points within the same group to show trends. (C) Dot plot of differential analysis. The plot shows the percentage difference and Log2FC of differentially expressed genes in each cell type. Each dot represents a gene's expression pattern in a specific cell type, with the vertical axis representing Log2FC and the horizontal axis representing the percentage difference.

Cell populations were annotated using established markers, with 12 distinct subclusters ultimately classified into six major cell types: oligodendrocyte, astrocyte, myeloid, neuron, fibroblast, and T cell (Figure 2B). We employed bubble and proportional bar charts to visually represent the established markers for these six cell types (Figure 2C,D). Subsequently, we performed an analysis of subpopulation differences and enrichment, utilizing ClusterGVis to generate a heat map and enrichment annotations reflecting the average expression levels of the cell subpopulations (Figure 3A). The results revealed that the DEGs associated with Fibroblasts were significantly enriched in pathways pertaining to extracellular matrix organization, extracellular structure organization, and cell‐substrate adhesion, among others.

### 3.3 Observed/Expected Ratio (Ro/e) Algorithm Calculates the Specificity of Cell Distribution

The expected value for single cell grouping was computed using the epitools: expected function, and Ro/e was subsequently determined. The primary advantage of the Ro/e method is its capacity to account for variations in total cell numbers across samples, thereby enabling direct identification of relative enrichment or depletion patterns of specific cell types. Deviations from 1 (where Ro/e > 1 indicates enrichment and Ro/e < 1 indicates depletion) directly demonstrate distribution specificity. The Ro/e method has been widely adopted in recent spatial biology research to evaluate cell‐type colocalization and distribution patterns, as evidenced by its application in studies of cancers such as glioblastoma and oral squamous cell carcinoma [36, 37]. The correlation of expected values between primary tumors and recurrent tumors within single cells was visualized using ggplot2 (Figure 3B), highlighting Fibroblast as the predominant cell type. The FindAllmarker function was used to find the DEGs of each cell subpopulation, with the screening conditions of avg_log2FC > 1 and p_val_adj < 0.05. The DEGs for each cell subpopulation were illustrated using the markerVolcano function, with the top five marker genes exhibiting the greatest fold change indicated (Figure 3C). The cell cluster under scrutiny demonstrated consistently high expression of classical fibroblast‐associated extracellular matrix genes, such as COL1A1/2, COL3A1, POSTN, and FN1, a profile strongly indicative of a fibroblastic identity. To further rule out potential misannotation with other cell types, we systematically evaluated the expression of established exclusion markers: characteristic pericyte markers (PDGFRB, RGS5) and glioma cell markers (SOX2, OLIG2) showed no significant expression. Based on this complementary molecular evidence, we are confident in the reliability of annotating this population as fibroblasts.

### 3.4 Random Survival Forest

**FIGURE 4:**
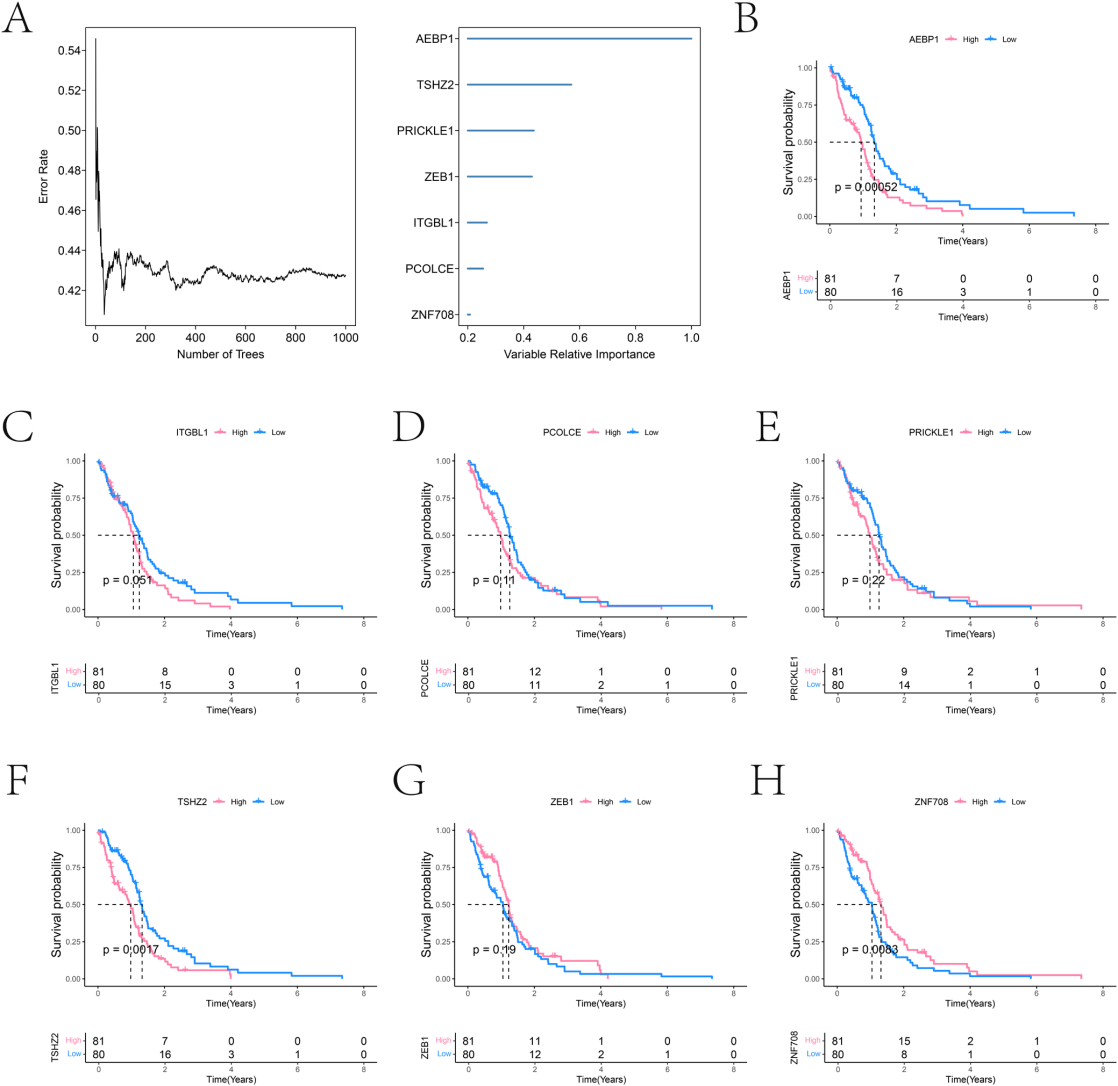
Random survival forest for key gene screening. (A) Left panel shows the trend of model error rate as the number of decision trees increases. The error rate stabilizes around 0.42 when the number of trees reaches approximately 800, indicating good model convergence. The right panel shows the variable relative importance of key genes, with AEBP1 (importance ≈1.0) and TSHZ2 (≈0.8) contributing the most to survival prediction. (B–H) Survival analysis grouped by median gene expression. Red lines represent the high‐expression group, and blue lines represent the low‐expression group. Significant differences in prognosis are observed for patients with different expression levels of AEBP1, ZNF708, and TSHZ2.

To enhance the identification of pivotal genes influencing glioma, we conducted a random survival forest analysis on the DEGs associated with fibroblasts. Internal validation performed with the out‐of‐bag (OOB) data from 1000 bootstrap replicates exhibited a convergent error rate curve, which signifies the model's good stability. We selected genes exhibiting a relative importance greater than 0.2 as the final markers and illustrated the hierarchical significance of the seven identified genes (Figure 4A). Moreover, we conducted a survival analysis on these seven genes and discovered that the survival associated with the AEBP1, ZNF708, and TSHZ2 genes was significant (Figure 4B–H).

### 3.5 Analysis of Chemotherapy Drug Sensitivity

**FIGURE 5:**
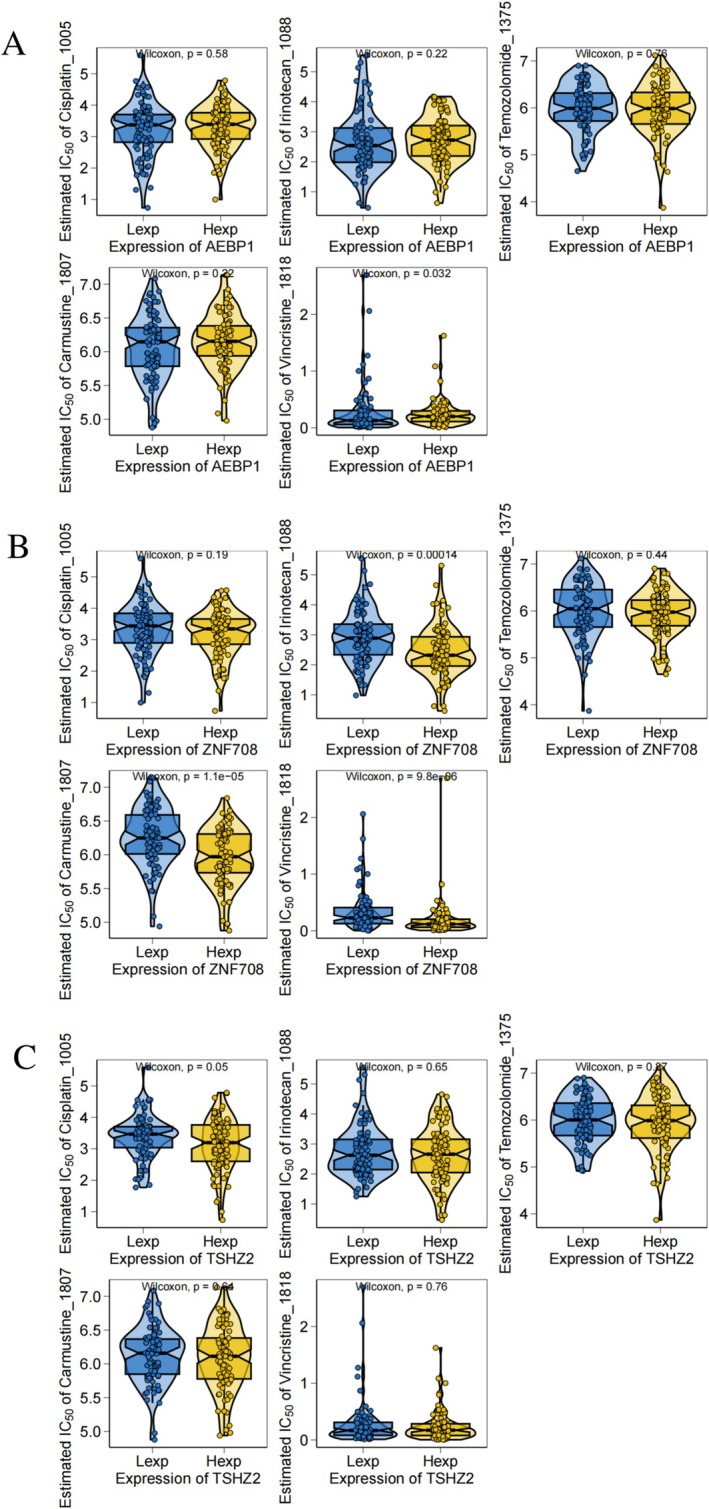
Drug sensitivity analysis. (A–C) Association analysis between expression levels and drug sensitivity. Violin plots show the distribution of half‐maximal inhibitory concentration (IC50) estimates for five chemotherapy drugs in high‐expression (HExp) and low‐expression (LExp) groups. Wilcoxon test is used to assess differences between groups.

Early‐stage gliomas are effectively treated through a combination of surgical intervention and chemotherapy. The present study utilizes drug sensitivity data from the GDSC database. We employed the R package “pRRophetic” to predict the chemotherapy sensitivity of individual tumor samples and to further examine the responsiveness of key genes to commonly utilized chemotherapy agents. Based on the drug sensitivity analysis, AEBP1 showed a significant correlation specifically with vincristine sensitivity, while ZNF708 exhibited highly significant associations with sensitivity to irinotecan, carmustine, and vincristine (Figure 5A,B); Similarly, TSHZ2 demonstrated a statistically significant association with cisplatin sensitivity (Figure 5C). In addition, we performed drug sensitivity analysis on several chemotherapeutic agents not routinely used in glioma to assess their potential therapeutic relevance (Figure S1A–C). These results highlight the potential of these genes to serve as selective predictors of response to specific chemotherapeutic agents.

### 3.6 Immune Infiltration

**FIGURE 6:**
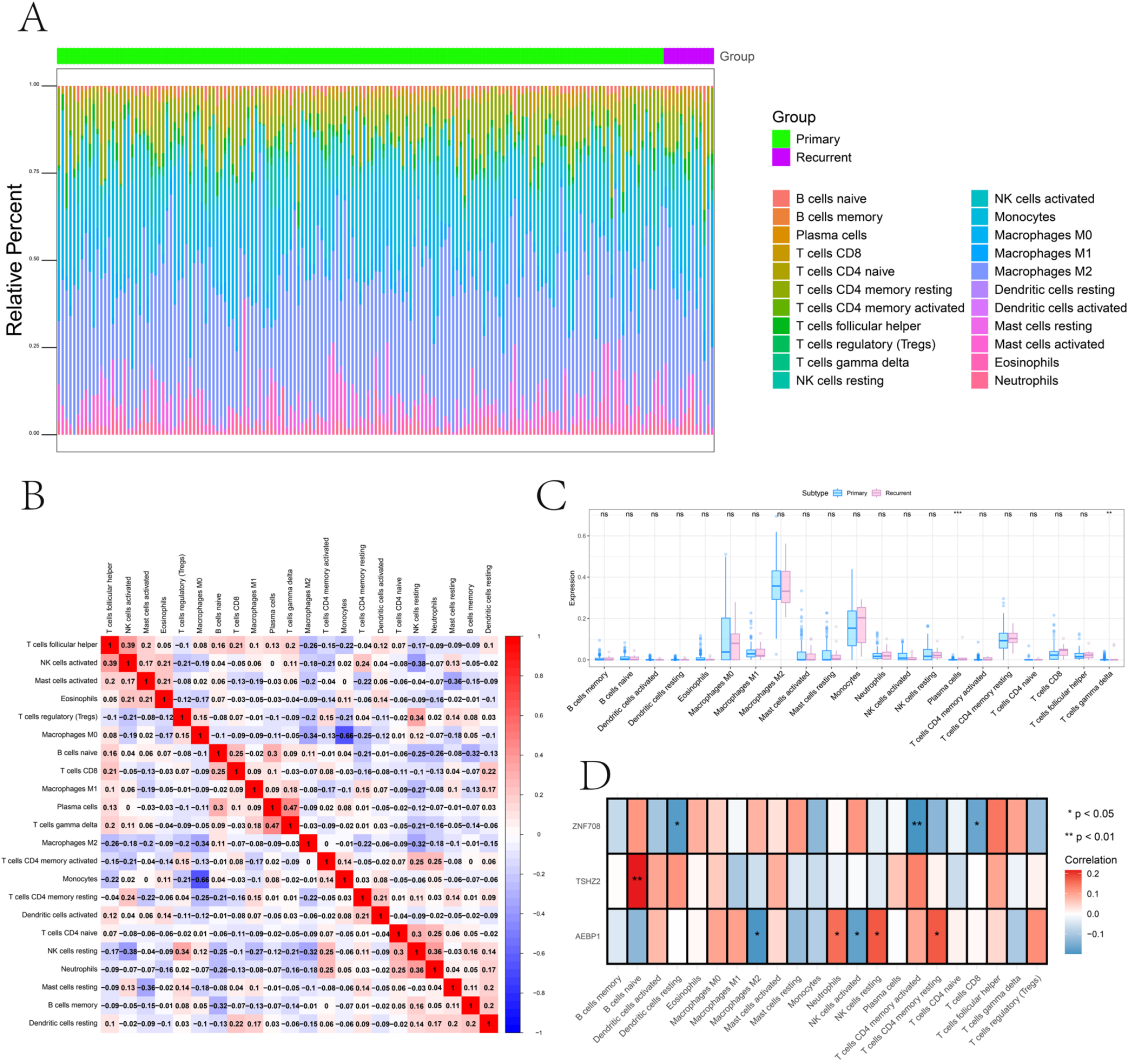
Immune infiltration. (A) Radar chart showing the proportion of immune cells in different groups (Primary, Recurrent) across samples. (B) Correlation heatmap of immune cells. Colors and correlation coefficient values indicate the strength of positive (red) and negative (blue) correlations. (C) Box plot comparing the expression levels of immune cells between Primary and Recurrent groups. (D) Heatmap showing statistical significance (*p* value) and expression trends of genes across different cells.

The microenvironment is primarily constituted of fibroblasts, immune cells, extracellular matrix components, various growth factors, inflammatory mediators, as well as distinct physical and chemical properties. Our research illustrates the distribution of immune infiltration levels and the correlations among immune cell types across different contexts (Figure 6A,B). The plasma cell levels in samples from the recurrent tumor group were notably elevated compared to those in the primary tumor group (Figure 6C). In our investigation of the association between pivotal genes and immune cell populations, we observed that AEBP1 exhibited a significant positive correlation with resting CD4 memory T cells, resting NK cells, and neutrophils. Conversely, it demonstrated a significant negative correlation with activated NK cells and M2 macrophages. Additionally, ZNF708 was found to have a significant negative correlation with CD8 T cells, activated CD4 memory T cells, and resting dendritic cells. Furthermore, TSHZ2 showed a significant positive correlation with naive B cells (Figure 6D).

Based on the ESTIMATE combined scores, we performed differential analysis between primary and recurrent tumor samples. The results showed that the recurrent group had significantly higher scores compared to the primary group, suggesting an increased overall infiltration of non‐tumor cellular components and/or greater heterogeneity in the tumor microenvironment of recurrent tumors (Figure S2).

### 3.7 GSEA Analysis

**FIGURE 7:**
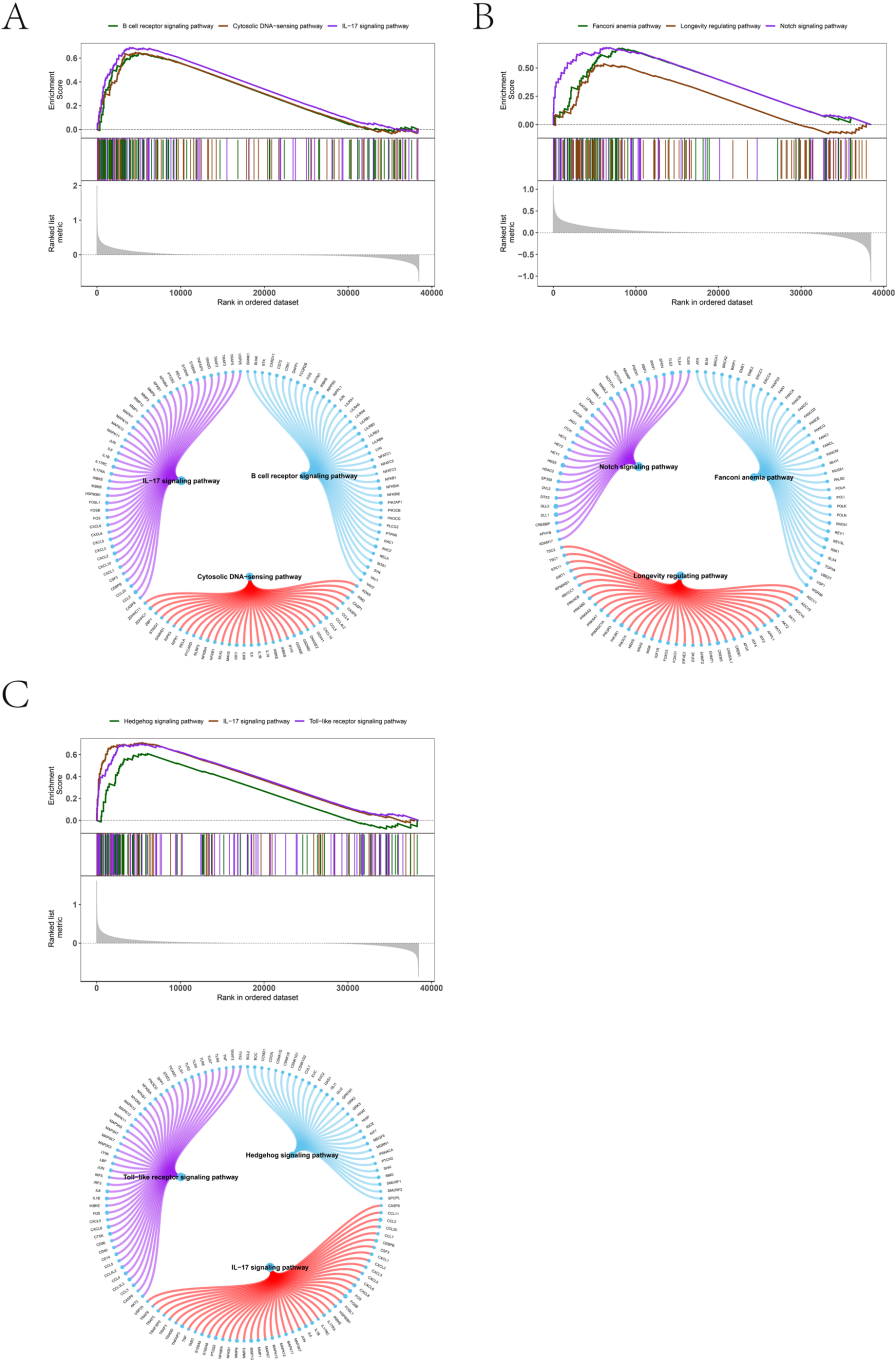
Gene set enrichment analysis (GSEA) analysis of key genes. (A–C) Integrated results of GSEA and interaction network analysis. The upper part shows GSEA results based on related signaling pathways: Each subplot displays the enrichment score (ES) of related signaling pathways (e.g., Wnt, Hippo, Notch). The *x*‐axis represents the rank of genes in the expression data, and the *y*‐axis represents the cumulative enrichment score. The enrichment level of signaling pathways reveals the biological processes potentially regulated by the target genes. The lower part shows the network diagram of target genes: A circular network diagram displays the relationships between target genes and their significantly related genes and signaling pathways. Colors indicate the direction of gene expression (red for upregulation, blue for downregulation), and the width of connecting lines reflects the strength of correlation.

Subsequently, we conducted an investigation into the specific signaling pathways associated with critical genes and examined the potential molecular mechanisms through which these genes influence disease progression. The results of the GSEA revealed that AEBP1 was significantly enriched in several signaling pathways, including the IL‐17 signaling pathway, the Cytosolic DNA‐sensing pathway, and the B cell receptor signaling pathway (Figure 7A), suggesting its potential role in modulating inflammatory responses and genomic instability within the tumor microenvironment. ZNF708 has been found to be significantly associated with various signaling pathways, including the Notch signaling pathway, the Fanconi anemia pathway, and the Longevity regulating pathway (Figure 7B), implicating this gene in the maintenance of cancer stem cells and DNA damage response processes. TSHZ2 is enriched in signaling pathways such as the Toll‐like receptor signaling pathway, IL‐17 signaling pathway, and Hedgehog signaling pathway (Figure 7C), indicating its involvement in both immune regulation and the activation of developmental pathways.

### 3.8 GSVA Analysis

**FIGURE 8:**
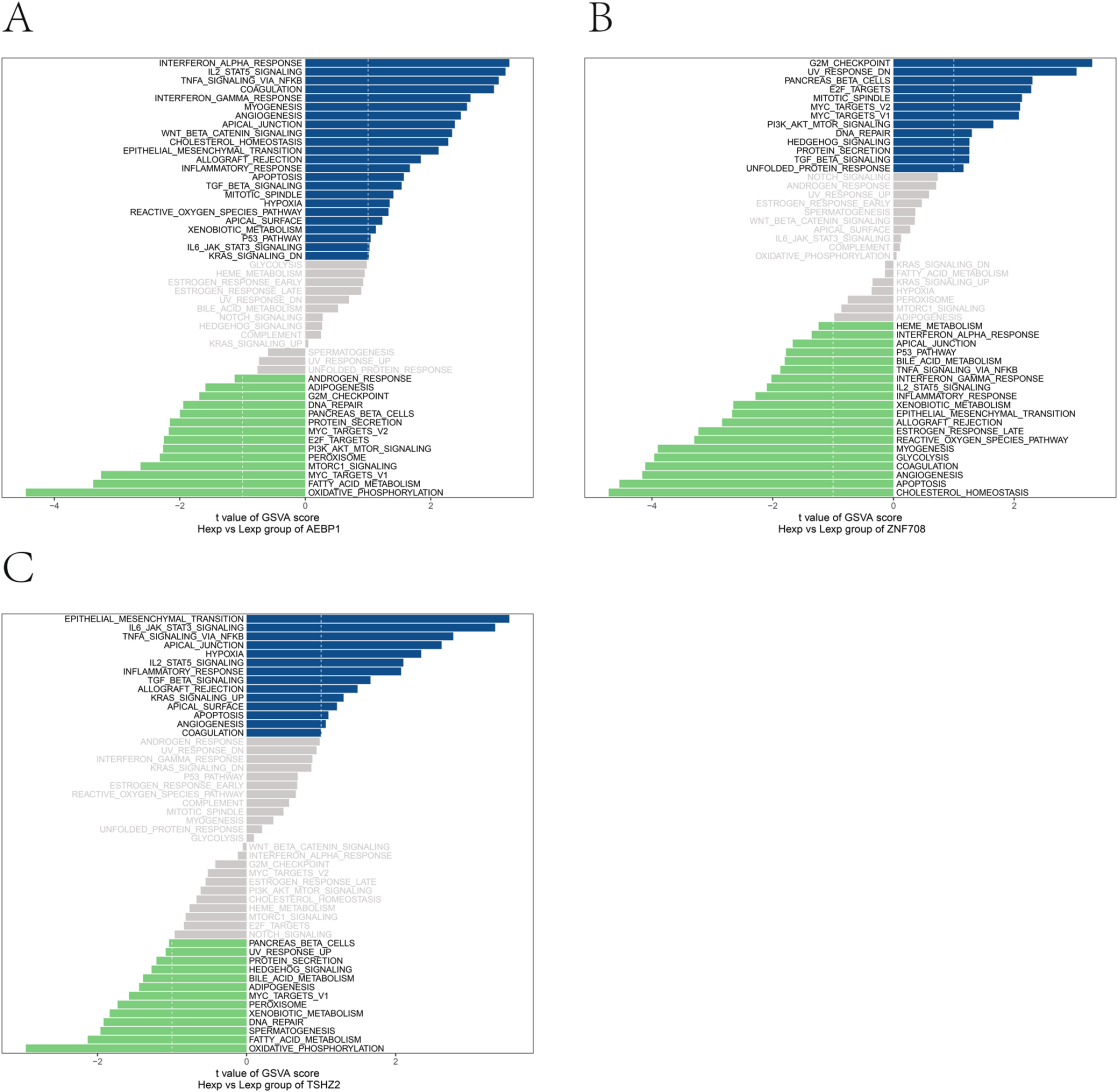
Gene set variation analysis (GSVA) analysis of key genes. (A–C) GSVA significant pathway bar chart of key genes. Each subgraph shows the sequencing results of functional pathways (up‐regulated and down‐regulated) that are significantly associated with the target gene, with blue representing up‐regulated pathways in which the gene is significantly involved, green representing down‐regulated pathways, and gray representing non‐significant pathways with low NES.

GSVA analysis revealed that AEBP1 exhibited significant enrichment in signaling pathways such as INTERFERON_ALPHA_RESPONSE and IL2_STAT5_SIGNALING (Figure 8A). ZNF708 was enriched in signaling pathways such as G2M_CHECKPOINT and UV_RESPONSE_DN (Figure 8B). Furthermore, TSHZ2 was found to be enriched in pathways associated with EPITHELIAL_MESENCHYMAL_TRANSITION and IL6_JAK_STAT3_SIGNALING (Figure 8C).

These findings indicate that these pivotal genes may influence tumor progression through their involvement in these specific signaling pathways.

We have now systematically ranked all pathway activity scores obtained through GSVA calculations, as detailed in Tables [Link], [Link]. In these tables, pathways are categorized into three groups: Group 1 represents significantly downregulated pathways, Group 2 indicates pathways with no significant change, and Group 3 corresponds to significantly upregulated pathways. In brief, a GSVA score > 0 suggests that high expression of the gene is positively correlated with the activity of a given pathway, whereas a score < 0 indicates a negative correlation between high gene expression and pathway activity.

### 3.9 Identification of Spatial Domains and RCTD Deconvolution

**FIGURE 9:**
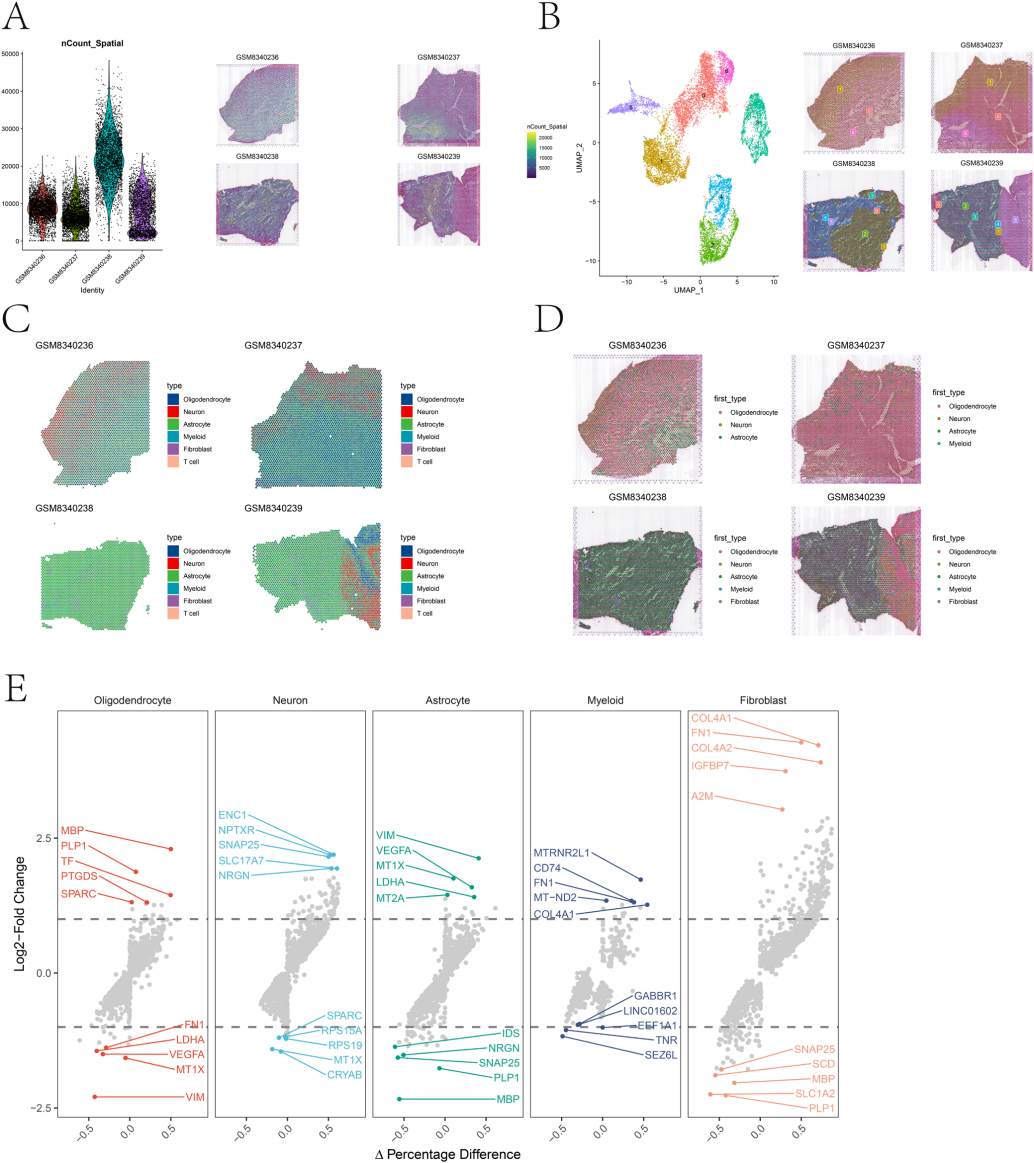
Dimensionality reduction, clustering, and subpopulation differential expression analysis of spatial transcriptomics data. (A) Quality assessment of spatial sequencing data for samples GSM8340236 to GSM8340239. Bar plots show the nCount_Spatial (spatial location gene count) for each sample, reflecting sequencing depth and data coverage. (B) UMAP dimensionality reduction visualization, with colors indicating different cell types, showing the spatial distribution heterogeneity of cell composition across samples. (C) Deconvolution analysis showing the predominant cell type for each spot. (D) Distribution of primary cell types (first_type) revealing microenvironment characteristics of each sample. (E) Differential expression analysis, with the *x*‐axis representing Log2FC and the *y*‐axis representing the percentage difference.

We sequentially analyzed four spatial transcriptome samples and assessed the distribution of UMI counts across these samples (Figure 9A). The data underwent a series of processes including standardization, normalization, PCA for linear dimension reduction, UMAP for nonlinear dimension reduction, and clustering with FindClusters, resulting in the identification of seven subgroups from the four samples (Figure 9B). We used the software package spacexr to combine single‐cell data to perform deconvolution analysis on the spatial transcriptome to determine the cell type with the largest proportion in each spot (Figure 9C,D). ST data were visualized using the SpatialFeaturePlot function from the Seurat package to generate spatial feature maps illustrating the distribution of various cell types across tissue sections (Figure S3). To verify the accuracy of deconvolution, we used the FindAllmarkers function to screen the key genes in each category (Figure 9E). The parameters applied for this screening included: logfc.threshold = 0, min.pct = 0.1, and only.pos = F.

### 3.10 Pathway Activity Analysis and Spatial Cell Interactions

PROGENy is adept at generating core gene sets for various biological pathways and assessing gene contribution weights, thereby improving the accuracy and efficacy of pathway activity evaluations. Furthermore, PROGENy is capable of identifying pathway nodes that exhibit significant alterations in lesions, which enhances the reliability of outcomes in biological research.

**FIGURE 10:**
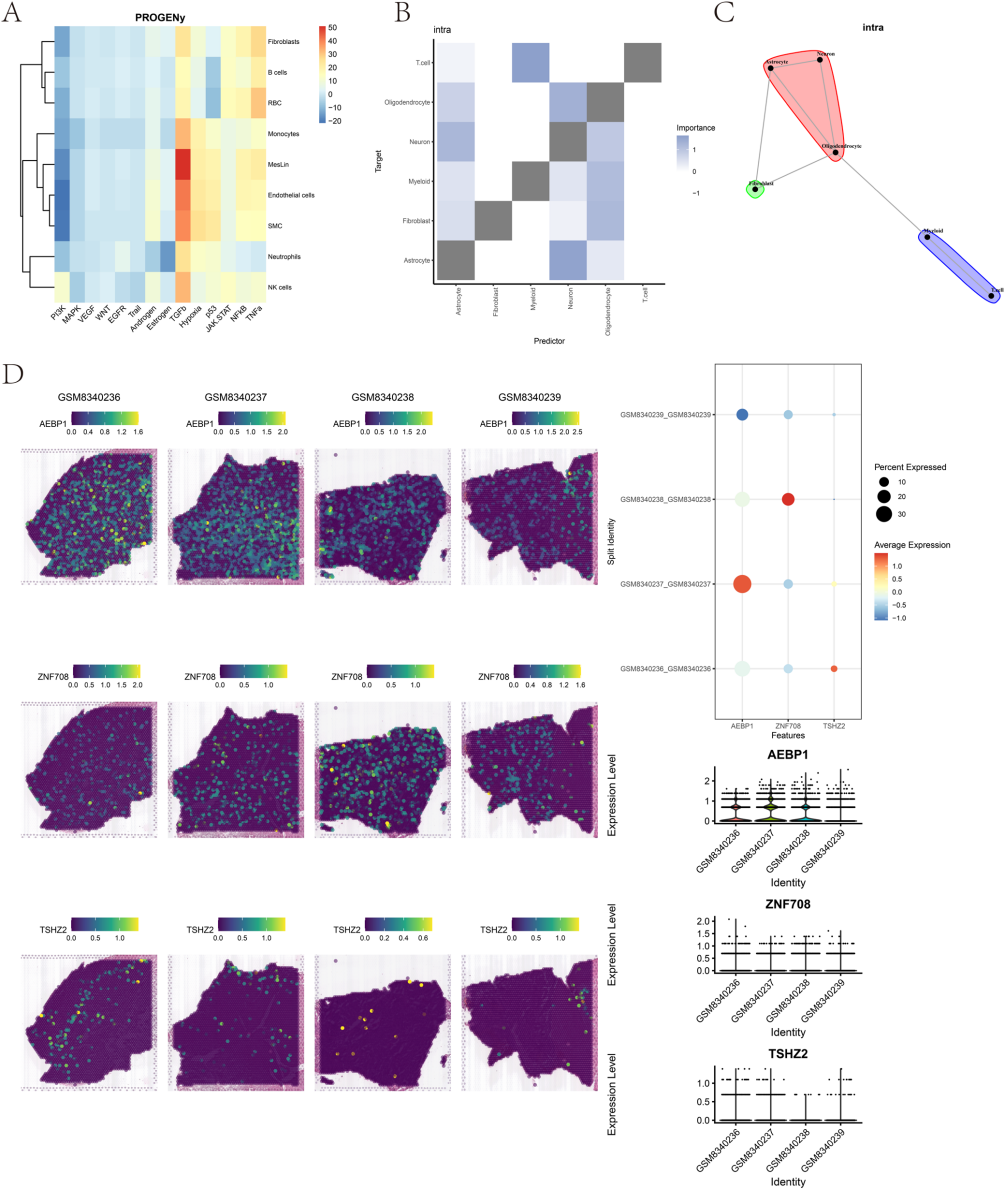
Regulatory network and expression characteristics of key genes and signaling pathways in the glioma microenvironment. (A) PROGENy pathway activity analysis showing the activity scores of 14 core signaling pathways in different cell types (e.g., fibroblasts, myeloid cells, T cells). (B, C) Heatmap and network diagram of spatial cell–cell interactions. The edge importance in the heatmap reflects the strength of cell–cell interactions; in the network diagram, more node connections indicate higher interactions. (D) Expression of key genes. Color gradients indicate expression levels.

This method, as utilized in a meningioma single‐cell transcriptomics study, is well‐established for pathway activity evaluations [38]. In our study, we employed PROGENy to assess the pathway activity of distinct cell subtypes within the spatial transcriptome, subsequently visualizing the results in a heat map (Figure 10A).

The findings indicated that the MesLin cell subtype exhibited elevated activity within the TGFb pathway. Additionally, we utilized MISTy to analyze both intracellular and intercellular relationships, thereby facilitating a more comprehensive understanding of marker interactions. By conducting cell interaction analyses on the annotated cell identities post‐deconvolution, we generated an intracellular interaction heat map and network diagram (Figure 10B,C). The analysis revealed that Oligodendrocyte and Astrocyte cells demonstrated heightened interaction levels. Lastly, we examined the expression of key genes within the spatial transcriptome, highlighting the expression abundance of AEBP1, ZNF708, and TSHZ2 across the four samples (Figure 10D). The results indicated that AEBP1 was predominantly expressed in GSM8340237, ZNF708 in GSM8340238, and TSHZ2 in GSM8340236.

## 4 Discussion

Our study integrates scRNA‐seq and ST to dissect mechanisms underlying glioma recurrence, providing a spatially resolved perspective that surpasses the limitations of prior single‐cell‐only analyses.

Unlike conventional scRNA‐seq which loses spatial context during tissue dissociation, ST preserves the native tissue organization while simultaneously capturing transcriptomic information. This unique capability allows researchers to investigate how cellular function and identity are shaped by their precise anatomical location and interactions with neighboring cells within intact tissue microenvironments. The spatial perspective fundamentally enhances our ability to investigate and ultimately target the complex cellular interactions underlying disease recurrence and therapeutic resistance in glioma.

Recurrence constitutes the primary factor contributing to the failure of glioma treatment, with approximately 90% of patients diagnosed with HCG experiencing a recurrence following therapeutic intervention [3]. To create therapies that can postpone tumor recurrence and effectively address recurrent tumors, it is essential to comprehend the mechanisms involved in glioma recurrence. Currently, factors contributing to glioma recurrence are thought to include incomplete surgical removal, the impact of the tumor microenvironment [39], and the tumor resistance to treatment [40]. However, the specifics are still not well understood. Additionally, due to various factors like drug resistance following glioma recurrence [41], the effectiveness of current treatment guidelines remains limited [42]. This underscores the necessity for further investigation into the mechanisms of glioma recurrence and its treatment.

A potentially effective approach is to focus on the elements of the tumor microenvironment (TME) that are more genomically stable. This research elucidated the significant role of fibroblasts and their associated key genes, including AEBP1, ZNF708, and TSHZ2, in the recurrence of glioma. Cancer‐associated fibroblasts (CAFs) represent a predominant component of the stromal environment within solid tumors and are integral to various aspects of tumor biology. They significantly influence tumor initiation, progression, metastasis, resistance to therapy, and evasion of the immune response by synthesizing a diverse array of extracellular matrix (ECM) proteins and regulatory molecules [43, 44, 45]. CAFs are integral to the remodeling of the ECM and the facilitation of immune evasion [46]. Consequently, CAFs represent viable candidates for the enhancement of cancer treatment methodologies. It is evident that numerous current therapeutic approaches impact the communication between CAFs and cancer cells, thereby altering the influence that CAFs exert on cancer cell behavior. For instance, BRAF inhibitors have been shown to activate stromal fibroblasts, which in turn may facilitate a compensatory mechanism in cancer cells that activates the ERK–MAPK pathway [47]. Numerous receptor tyrosine kinase inhibitors that are being developed show some effectiveness against FGF and PDGF receptors, which can influence fibroblast activity [48, 49]. Besides, researchers found that fibrotic niches developing after treatment enhance the recurrence of glioma. This fibrotic response to treatment is driven by fibroblast‐like cells originating from perivascular regions, which operate through transforming growth factor β (TGF‐β) signaling and neuroinflammatory activation [50]. Collectively, these findings underscore the potential of targeting fibroblasts as a promising therapeutic strategy.

Finding and validating the key genes of glioma recurrence is of great significance for improving prognosis management. This research indicates that glioma patients exhibiting low levels of TSHZ2 and AEBP1 expression demonstrate an increased overall survival rate. This is the opposite of ZNF708. AEBP1 has been demonstrated to have a strong connection to cancer advancement [51, 52]. Studies by Cheng et al. demonstrated that elevated AEBP1 expression correlates with poor prognosis in glioma [53]. According to reports, AEBP1 might enhance the growth and advancement of glioma cells in glioblastoma by activating the NF‐κB pathway and its associated targets, which is associated with immunity in tumors [51]. Furthermore, the expression pattern of AEBP1 may influence the infiltration of immune cells [54]. Consequently, AEBP1 emerges as a potential prognostic biomarker related to immune response in the context of glioma recurrence. In line with existing literature, our data corroborate the significant correlation between AEBP1 overexpression and unfavorable patient outcomes in glioma, thereby underscoring the consistency and reliability of our findings. TSHZ2 is a member of the teashirt C2H2‐type zinc‐finger protein family, which is known for its role as transcriptional repressors in various developmental processes. Currently, the functional implications of TSHZ2 are primarily associated with lung adenocarcinoma, breast cancer, Alzheimer's disease, congenital pelvi‐ureteric junction obstruction, and craniosynostosis [55, 56, 57]. ZNF708 remains poorly characterized, with only one study linking it to breast cancer reported so far [58]. To our knowledge, no prior studies have reported roles for TSHZ2 or ZNF708 in glioma. Our work is the first to identify them as potential drivers of glioma recurrence.

Furthermore, pivotal genes play a crucial role in informing personalized treatment strategies. The expression levels of these key genes demonstrated a significant correlation with the sensitivity to various chemotherapeutic agents, such as Vincristine, Gemcitabine, Etoposide, and Cytarabine, thereby presenting potential targets for the tailored treatment of relapsed glioma.

GSEA, GSVA, and PROGENy analyses may yield partially overlapping yet occasionally inconsistent results. Such variability is inherent to their distinct methodological frameworks; however, these approaches should be interpreted as complementary rather than contradictory. The observed differences reflect their unique analytical perspectives: while GSEA emphasizes phenotype‐associated pathway enrichment, GSVA captures inter‐sample heterogeneity in pathway activity, and PROGENy provides focused inference of signaling pathway perturbations. Collectively, these methods offer a multi‐faceted understanding of biological processes, enhancing the robustness and comprehensiveness of our functional insights into the roles of AEBP1, ZNF708, and TSHZ2 in glioma progression. AEBP1 was consistently associated with inflammatory responses and antiviral immunity in both GSEA and GSVA analyses, suggesting its potential role in influencing glioma progression through modulation of the tumor immune microenvironment. The pathways enriched for ZNF708 indicate its involvement in DNA damage repair and cell cycle regulation. TSHZ2 expression correlates with tumor progression and cellular plasticity, contributing to enhanced invasive and metastatic potential. PROGENy analysis further complemented these findings at the cellular subpopulation level. By systematically comparing overlapping and unique pathway findings, we will strengthen the biological interpretation of our results and provide a more nuanced perspective on the functional implications of the identified genes.

Taken together, the objective of this research is to identify and validate the critical genes associated with glioma recurrence utilizing scRNA‐seq and ST methodologies. Additionally, the study seeks to explore potential avenues for enhancing the prognostic management of patients experiencing recurrent glioma.

## 5 Limitations

Despite these promising findings, it also recognizes specific limitations. First, in the TCGA database, the recurrence glioma group contains only 13 samples. In the GEO database, the single‐cell dataset includes only 42 recurrence samples, and the spatial transcriptome data file contains only four glioma samples for spatial transcriptome analysis. The sample sizes for the single‐cell and spatial transcriptome data are relatively small. Accordingly, expanding the cohort will be a key objective in future research. Secondly, the roles of the three key genes—AEBP1, ZNF708, and TSHZ2—in glioma recurrence and drug sensitivity predictions were not validated through in vitro or in vivo experiments. Independent validation will be an important focus of our future work. Finally, while our study has identified promising therapeutic candidates, their translational potential requires further investigation through functional validation of drug efficacy and assessment of clinical applicability.

## Author Contributions

Data management: Lei Qiu, Yinjiao Fei, and Jiaxuan Ding. Formal analysis: Kexin Shi and Weilin Xu. Survey: Yuchen Zhu, Kexin Shi, Gefei Jiang, and Weilin Xu. Methodology: Lei Qiu. Project Management: Shu Zhou. Resources: Yuandong Cao. Software: Yinjiao Fei, Yuchen Zhu, Gefei Jiang, and Xingjian Sun. Supervision: Yuandong Cao. Validation: Yinjiao Fei. Visualization: Jiaxuan Ding, Lei Qiu, and Jinyan Luo. Writing – original draft: Lei Qiu, Yinjiao Fei, and Jiaxuan Ding. Writing – review and editing: Yuandong Cao, Weilin Xu, and Shu Zhou. All authors contributed to the article and approved the submitted version.

## Funding

The authors have nothing to report.

## Ethics Statement

This study utilized publicly available, de‐identified datasets from TCGA and GEO. The Ethics Committee of the First Affiliated Hospital with Nanjing Medical University reviewed and granted an exemption for ethical approval.

## Consent

All authors approved the final manuscript and the submission to this journal.

## Conflicts of Interest

The authors declare no conflicts of interest.

## Supporting information


**Figure S1:** Drug sensitivity analysis. (A–C) Association analysis between expression levels and drug sensitivity. Violin plots show the distribution of half‐maximal inhibitory concentration (IC50) estimates for six chemotherapy drugs in high‐expression (HExp) and low‐expression (LExp) groups. Wilcoxon test is used to assess differences between groups.

**Figure S2:** ESTIMATE combined scores across samples.

**Figure S3:** Spatial cell type mapping using RCTD. The spatial distribution of major cell types across tissue sections is visualized, with color intensity reflecting the relative abundance of each cell type at every spatial spot. This reveals the specific localization and spatial heterogeneity of oligodendrocytes, neurons, astrocytes, myeloid cells, fibroblasts, and T cells.

**Table S1:** GSVA enrichment scores for AEBP1‐associated signaling pathways.

**Table S2:** GSVA enrichment scores for ZNF708‐associated signaling pathways.

**Table S3:** GSVA enrichment scores for TSHZ2‐associated signaling pathways.

## Data Availability

The data that support the findings of this study are available in GEO at https://www.ncbi.nlm.nih.gov/geo/, reference number GSE174554 and GSE270355. These data were derived from the following resources available in the public domain: GEO, https://www.ncbi.nlm.nih.gov/geo/.
