# Supplementary figures and images for: Unraveling the Molecular Mechanisms of Glioma Recurrence: A Study Integrating Single‐Cell and Spatial Transcriptomics
Source: Ann Clin Transl Neurol. 2026 Jan 6;13(6):1160–78. doi: 10.1002/acn3.70306 (PMC13251444; doi:10.1002/acn3.70306)

A

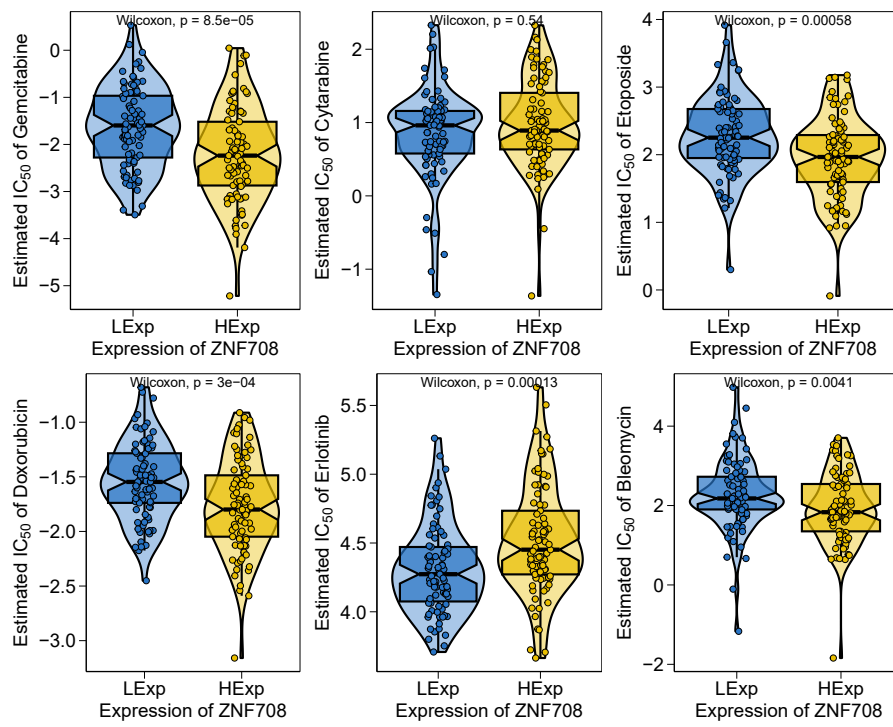

B

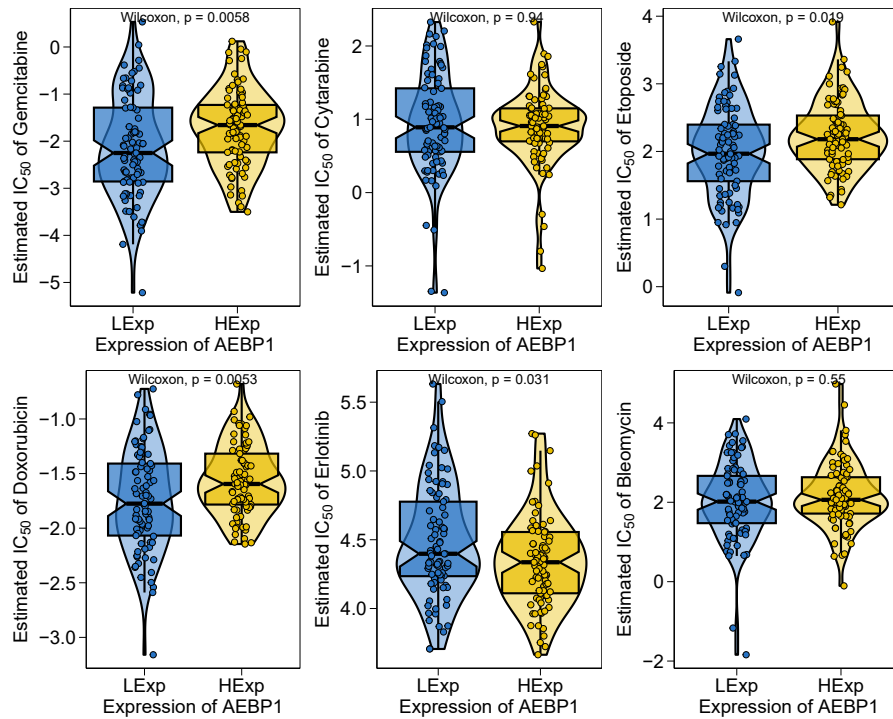

C

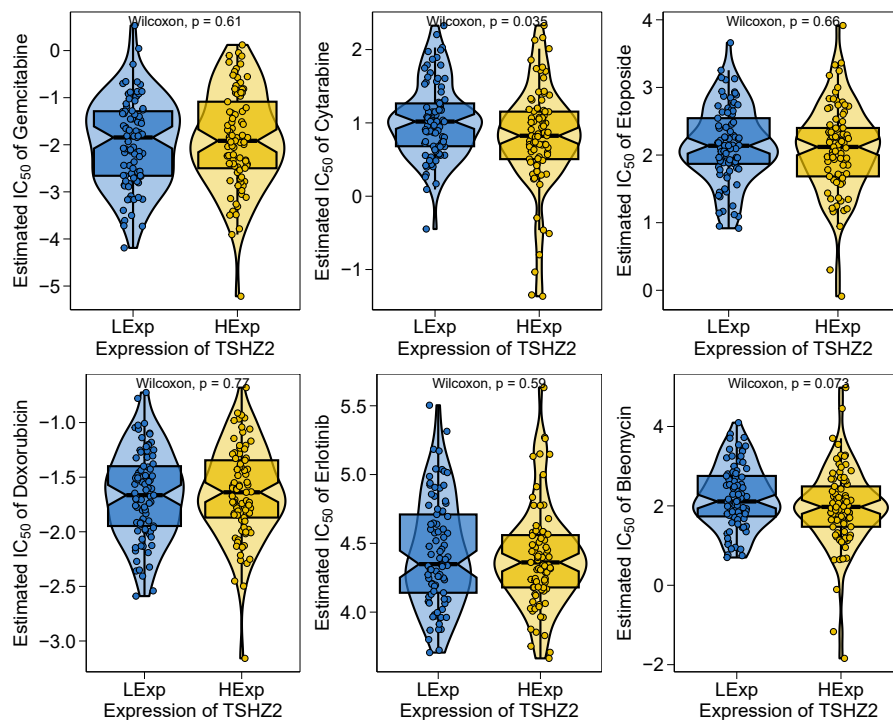

Supplement: Supplementary file 1 — Figure S1: Drug sensitivity analysis. (A–C) Association analysis between expression levels and drug sensitivity. Violin plots show the distribution of half‐maximal inhibitory concentration (IC50) estimates for six chemotherapy drugs in high‐expression (HExp) and low‐expression (LExp) groups. Wilcoxon test is used to assess differences between groups. [file ACN3-13-1160-s004.pdf]

Subtype Primary Recurrent

Expression

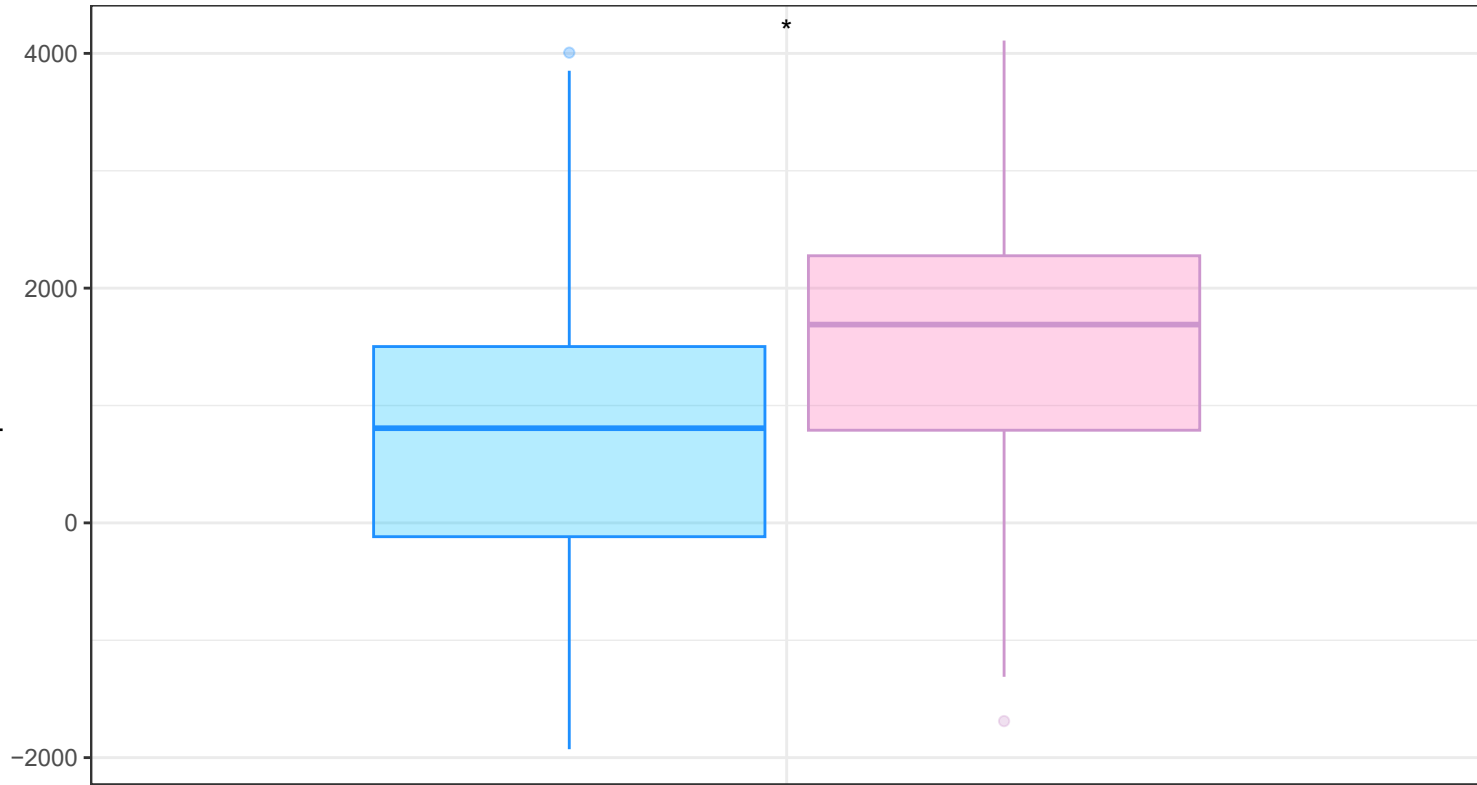

ESTIMATEScore

Supplement: Supplementary file 2 — Figure S2: ESTIMATE combined scores across samples. [file ACN3-13-1160-s006.pdf]

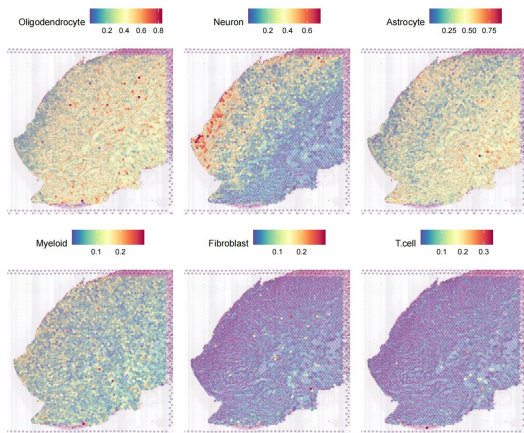

GSM8340236

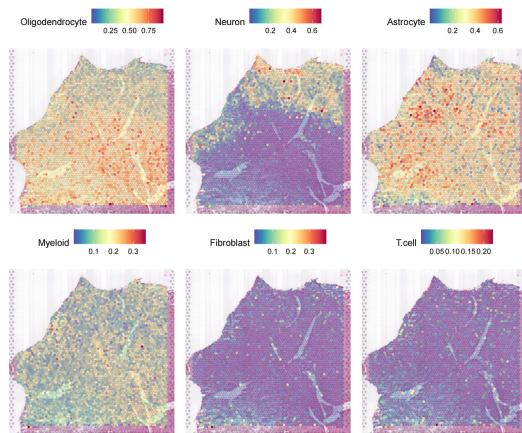

GSM8340237

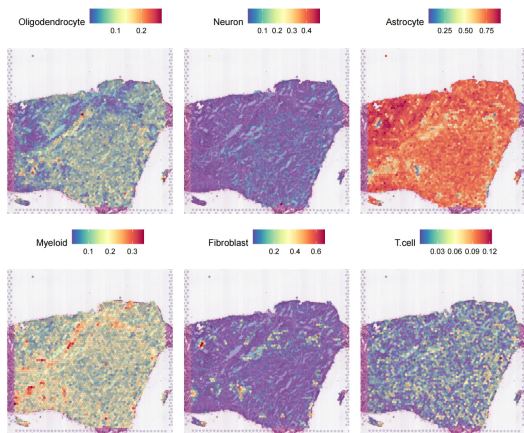

GSM8340238

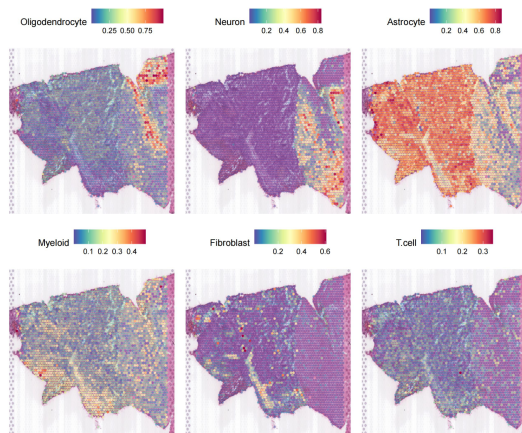

GSM8340239

Supplement: Supplementary file 3 — Figure S3: Spatial cell type mapping using RCTD. The spatial distribution of major cell types across tissue sections is visualized, with color intensity reflecting the relative abundance of each cell type at every spatial spot. This reveals the specific localization and spatial heterogeneity of oligodendrocytes, neurons, astrocytes, myeloid cells, fibroblasts, and T cells. [file ACN3-13-1160-s002.pdf]
