# Supplementary material for: Unraveling the Molecular Mechanisms of Glioma Recurrence: A Study Integrating Single‐Cell and Spatial Transcriptomics
Source: Ann Clin Transl Neurol. 2026 Jan 6;13(6):1160–78. doi: 10.1002/acn3.70306 (PMC13251444; doi:10.1002/acn3.70306)
Supplement: Supplementary file 4 — Table S1: GSVA enrichment scores for AEBP1‐associated signaling pathways. [file ACN3-13-1160-s001.docx]

Supplementary Table 1. GSVA enrichment scores for AEBP1-associated signaling pathways.

| ID | Score | Group |
| --- | --- | --- |
| OXIDATIVE_PHOSPHORYLATION | -4.4538145 | 1 |
| FATTY_ACID_METABOLISM | -3.3800834 | 1 |
| MYC_TARGETS_V1 | -3.2534789 | 1 |
| MTORC1_SIGNALING | -2.6252629 | 1 |
| PEROXISOME | -2.3179504 | 1 |
| PI3K_AKT_MTOR_SIGNALING | -2.2677807 | 1 |
| E2F_TARGETS | -2.2536099 | 1 |
| MYC_TARGETS_V2 | -2.1760637 | 1 |
| PROTEIN_SECRETION | -2.1578222 | 1 |
| PANCREAS_BETA_CELLS | -1.9979631 | 1 |
| DNA_REPAIR | -1.9454127 | 1 |
| G2M_CHECKPOINT | -1.6899019 | 1 |
| ADIPOGENESIS | -1.5931437 | 1 |
| ANDROGEN_RESPONSE | -1.1266825 | 1 |
| UNFOLDED_PROTEIN_RESPONSE | -0.7629693 | 2 |
| UV_RESPONSE_UP | -0.7373284 | 2 |
| SPERMATOGENESIS | -0.5932805 | 2 |
| KRAS_SIGNALING_UP | 0.04671155 | 2 |
| COMPLEMENT | 0.25023064 | 2 |
| HEDGEHOG_SIGNALING | 0.26827355 | 2 |
| NOTCH_SIGNALING | 0.27623381 | 2 |
| BILE_ACID_METABOLISM | 0.5210394 | 2 |
| UV_RESPONSE_DN | 0.69696888 | 2 |
| ESTROGEN_RESPONSE_LATE | 0.89132772 | 2 |
| ESTROGEN_RESPONSE_EARLY | 0.92203912 | 2 |
| HEME_METABOLISM | 0.94704989 | 2 |
| GLYCOLYSIS | 0.97935011 | 2 |
| KRAS_SIGNALING_DN | 1.01411053 | 3 |
| IL6_JAK_STAT3_SIGNALING | 1.02202057 | 3 |
| P53_PATHWAY | 1.04137108 | 3 |
| XENOBIOTIC_METABOLISM | 1.12520507 | 3 |
| APICAL_SURFACE | 1.22797776 | 3 |
| REACTIVE_OXYGEN_SPECIES_PATHWAY | 1.32584827 | 3 |
| HYPOXIA | 1.34399881 | 3 |
| MITOTIC_SPINDLE | 1.40381826 | 3 |
| TGF_BETA_SIGNALING | 1.53585133 | 3 |
| APOPTOSIS | 1.56994395 | 3 |
| INFLAMMATORY_RESPONSE | 1.66593009 | 3 |
| ALLOGRAFT_REJECTION | 1.8415375 | 3 |
| EPITHELIAL_MESENCHYMAL_TRANSITION | 2.12419471 | 3 |
| CHOLESTEROL_HOMEOSTASIS | 2.2780641 | 3 |
| WNT_BETA_CATENIN_SIGNALING | 2.3386182 | 3 |
| APICAL_JUNCTION | 2.38376577 | 3 |
| ANGIOGENESIS | 2.47850357 | 3 |
| MYOGENESIS | 2.57584774 | 3 |
| INTERFERON_GAMMA_RESPONSE | 2.63258802 | 3 |
| COAGULATION | 3.00705686 | 3 |
| TNFA_SIGNALING_VIA_NFKB | 3.08165578 | 3 |
| IL2_STAT5_SIGNALING | 3.19075161 | 3 |
| INTERFERON_ALPHA_RESPONSE | 3.25170073 | 3 |

1. Significantly Downregulated Pathways;
2. No Significant Pathways;
3. Significantly Upregulated Pathways.

Score > 0: High expression of gene is positively correlated with the activity of pathway ID.

Score < 0: High expression of gene is negatively correlated with the activity of pathway ID.
