# Supplementary material for: Unraveling the Molecular Mechanisms of Glioma Recurrence: A Study Integrating Single‐Cell and Spatial Transcriptomics
Source: Ann Clin Transl Neurol. 2026 Jan 6;13(6):1160–78. doi: 10.1002/acn3.70306 (PMC13251444; doi:10.1002/acn3.70306)
Supplement: Supplementary file 5 — Table S2: GSVA enrichment scores for ZNF708‐associated signaling pathways. [file ACN3-13-1160-s005.docx]

Supplementary Table 2. GSVA enrichment scores for ZNF708-associated signaling pathways.

| ID | score | group |
| --- | --- | --- |
| CHOLESTEROL_HOMEOSTASIS | -4.716646 | 1 |
| APOPTOSIS | -4.538752 | 1 |
| ANGIOGENESIS | -4.1593018 | 1 |
| COAGULATION | -4.111415 | 1 |
| GLYCOLYSIS | -3.9629761 | 1 |
| MYOGENESIS | -3.9030963 | 1 |
| REACTIVE_OXYGEN_SPECIES_PATHWAY | -3.2989843 | 1 |
| ESTROGEN_RESPONSE_LATE | -3.2295817 | 1 |
| ALLOGRAFT_REJECTION | -2.8380127 | 1 |
| EPITHELIAL_MESENCHYMAL_TRANSITION | -2.6731709 | 1 |
| XENOBIOTIC_METABOLISM | -2.6527287 | 1 |
| INFLAMMATORY_RESPONSE | -2.2853989 | 1 |
| IL2_STAT5_SIGNALING | -2.0965116 | 1 |
| INTERFERON_GAMMA_RESPONSE | -2.0210316 | 1 |
| TNFA_SIGNALING_VIA_NFKB | -1.8738053 | 1 |
| BILE_ACID_METABOLISM | -1.8000982 | 1 |
| P53_PATHWAY | -1.7809893 | 1 |
| APICAL_JUNCTION | -1.6690079 | 1 |
| INTERFERON_ALPHA_RESPONSE | -1.3538324 | 1 |
| HEME_METABOLISM | -1.2403938 | 1 |
| ADIPOGENESIS | -0.9787779 | 2 |
| MTORC1_SIGNALING | -0.8611401 | 2 |
| PEROXISOME | -0.7553821 | 2 |
| HYPOXIA | -0.3613253 | 2 |
| KRAS_SIGNALING_UP | -0.3462855 | 2 |
| FATTY_ACID_METABOLISM | -0.1403091 | 2 |
| KRAS_SIGNALING_DN | -0.1377938 | 2 |
| OXIDATIVE_PHOSPHORYLATION | 0.05090042 | 2 |
| COMPLEMENT | 0.10757749 | 2 |
| IL6_JAK_STAT3_SIGNALING | 0.12435324 | 2 |
| APICAL_SURFACE | 0.27713961 | 2 |
| WNT_BETA_CATENIN_SIGNALING | 0.35337884 | 2 |
| SPERMATOGENESIS | 0.36341759 | 2 |
| ESTROGEN_RESPONSE_EARLY | 0.46920366 | 2 |
| UV_RESPONSE_UP | 0.58906233 | 2 |
| ANDROGEN_RESPONSE | 0.70760194 | 2 |
| NOTCH_SIGNALING | 0.73242564 | 2 |
| UNFOLDED_PROTEIN_RESPONSE | 1.16107078 | 3 |
| TGF_BETA_SIGNALING | 1.25498645 | 3 |
| PROTEIN_SECRETION | 1.25751579 | 3 |
| HEDGEHOG_SIGNALING | 1.25940081 | 3 |
| DNA_REPAIR | 1.29832662 | 3 |
| PI3K_AKT_MTOR_SIGNALING | 1.65432294 | 3 |
| MYC_TARGETS_V1 | 2.07887355 | 3 |
| MYC_TARGETS_V2 | 2.09861227 | 3 |
| MITOTIC_SPINDLE | 2.12846778 | 3 |
| E2F_TARGETS | 2.28246053 | 3 |
| PANCREAS_BETA_CELLS | 2.30232712 | 3 |
| UV_RESPONSE_DN | 3.03253252 | 3 |
| G2M_CHECKPOINT | 3.29185312 | 3 |

1. Significantly Downregulated Pathways;
2. No Significant Pathways;
3. Significantly Upregulated Pathways.

Score > 0: High expression of gene is positively correlated with the activity of pathway ID.

Score < 0: High expression of gene is negatively correlated with the activity of pathway ID.
