# Supplementary material for: Unraveling the Molecular Mechanisms of Glioma Recurrence: A Study Integrating Single‐Cell and Spatial Transcriptomics
Source: Ann Clin Transl Neurol. 2026 Jan 6;13(6):1160–78. doi: 10.1002/acn3.70306 (PMC13251444; doi:10.1002/acn3.70306)
Supplement: Supplementary file 6 — Table S3: GSVA enrichment scores for TSHZ2‐associated signaling pathways. [file ACN3-13-1160-s003.docx]

Supplementary Table 3. GSVA enrichment scores for TSHZ2-associated signaling pathways.

| ID | score | group |
| --- | --- | --- |
| OXIDATIVE_PHOSPHORYLATION | -2.9617297 | 1 |
| FATTY_ACID_METABOLISM | -2.1317453 | 1 |
| SPERMATOGENESIS | -1.9601921 | 1 |
| DNA_REPAIR | -1.919173 | 1 |
| XENOBIOTIC_METABOLISM | -1.8376122 | 1 |
| PEROXISOME | -1.7303774 | 1 |
| MYC_TARGETS_V1 | -1.5781566 | 1 |
| ADIPOGENESIS | -1.4415435 | 1 |
| BILE_ACID_METABOLISM | -1.3895379 | 1 |
| HEDGEHOG_SIGNALING | -1.275137 | 1 |
| PROTEIN_SECRETION | -1.2093861 | 1 |
| UV_RESPONSE_UP | -1.0857029 | 1 |
| PANCREAS_BETA_CELLS | -1.0408919 | 1 |
| NOTCH_SIGNALING | -0.9643018 | 2 |
| E2F_TARGETS | -0.8409324 | 2 |
| MTORC1_SIGNALING | -0.8175052 | 2 |
| HEME_METABOLISM | -0.7658357 | 2 |
| CHOLESTEROL_HOMEOSTASIS | -0.6719295 | 2 |
| PI3K_AKT_MTOR_SIGNALING | -0.6158296 | 2 |
| ESTROGEN_RESPONSE_LATE | -0.5504319 | 2 |
| MYC_TARGETS_V2 | -0.5195871 | 2 |
| G2M_CHECKPOINT | -0.4189112 | 2 |
| INTERFERON_ALPHA_RESPONSE | -0.1180639 | 2 |
| WNT_BETA_CATENIN_SIGNALING | -0.0508493 | 2 |
| GLYCOLYSIS | 0.09872052 | 2 |
| UNFOLDED_PROTEIN_RESPONSE | 0.20804146 | 2 |
| MYOGENESIS | 0.35959021 | 2 |
| MITOTIC_SPINDLE | 0.49486054 | 2 |
| COMPLEMENT | 0.57062313 | 2 |
| REACTIVE_OXYGEN_SPECIES_PATHWAY | 0.66015665 | 2 |
| ESTROGEN_RESPONSE_EARLY | 0.67932246 | 2 |
| P53_PATHWAY | 0.68441232 | 2 |
| KRAS_SIGNALING_DN | 0.86929945 | 2 |
| INTERFERON_GAMMA_RESPONSE | 0.88225691 | 2 |
| UV_RESPONSE_DN | 0.93941037 | 2 |
| ANDROGEN_RESPONSE | 0.98179318 | 2 |
| COAGULATION | 1.00468741 | 3 |
| ANGIOGENESIS | 1.06287418 | 3 |
| APOPTOSIS | 1.09851575 | 3 |
| APICAL_SURFACE | 1.21193552 | 3 |
| KRAS_SIGNALING_UP | 1.30229036 | 3 |
| ALLOGRAFT_REJECTION | 1.49005707 | 3 |
| TGF_BETA_SIGNALING | 1.66221465 | 3 |
| INFLAMMATORY_RESPONSE | 2.07298862 | 3 |
| IL2_STAT5_SIGNALING | 2.10362006 | 3 |
| HYPOXIA | 2.34250483 | 3 |
| APICAL_JUNCTION | 2.61720781 | 3 |
| TNFA_SIGNALING_VIA_NFKB | 2.77349554 | 3 |
| IL6_JAK_STAT3_SIGNALING | 3.3345684 | 3 |
| EPITHELIAL_MESENCHYMAL_TRANSITION | 3.52515184 | 3 |

1. Significantly Downregulated Pathways;
2. No Significant Pathways;
3. Significantly Upregulated Pathways.

Score > 0: High expression of gene is positively correlated with the activity of pathway ID.

Score < 0: High expression of gene is negatively correlated with the activity of pathway ID.
